# IMAGINE study protocol of a clinical trial: a multi-center, investigator-blinded, randomized, 36-month, parallel-group to compare the effectiveness of motivational interview in rehabilitation of older stroke survivors

**DOI:** 10.1186/s12877-020-01694-6

**Published:** 2020-09-04

**Authors:** Neus Gual, Laura Mónica Pérez, Carmina Castellano-Tejedor, Pilar Lusilla-Palacios, Judith Castro, Luís Soto-Bagaria, Laura Coll-Planas, Marta Roqué, Ana Belen Vena, Benito Fontecha, Jose M. Santiago, Eva Månsson Lexell, Carlos Chiatti, Susanne Iwarsson, Marco Inzitari

**Affiliations:** 1 REFiT BCN Research Group-Parc Sanitari Pere Virgili-Vall d’Hebron Institute of Research, Barcelona, Spain; 2 Autonomous University of Barcelona, Bellaterra, Spain; 3 Psychiatry Department, University Hospital Vall d’Hebron, Barcelona, Spain; 4 Fundació Salut i Envelliment (Foundation on Health and Ageing), Universitat Autònoma de Barcelona, Barcelona, Spain; 5 Institute of Biomedical Research (IIB Sant Pau), Barcelona, Spain; 6 University Hospital Santa Maria, Lleida, Spain; 7 Hospital General de l’Hospitalet (Consorci Sanitari Integral), Hospitalet de Llobregat, Spain; 8 Department of Health Sciences, Lund University, Lund, Sweden; 9 Department of Neurology and Rehabilitation Medicine, Skåne University Hospital, Lund-Malmö, Sweden

**Keywords:** Stroke, Geriatrics, Rehabilitation, Functional recovery, Physical activity, Motivational interviewing, Adherence, Clinical trial

## Abstract

**Background:**

Rehabilitation pathways are crucial to reduce stroke-related disability. Motivational Interviewing (MI), as a person-centered complex intervention, aimed to empower and motivate, and could be a resource to improve rehabilitation outcomes for older stroke survivors. The IMAGINE project aims to assess the impact of MI, as a complement to standard geriatric rehabilitation, on functional improvement at 30 days after admission, compared to standard geriatric rehabilitation alone, in persons admitted to geriatric rehabilitation after a stroke. Secondary objectives include assessing the impact of MI on physical activity and performance, self-efficacy, safety, cost-utility, participants’ experiences and functional status at 3 months.

**Methods:**

We will conduct a multicenter randomized clinical trial in three geriatric rehabilitation hospitals in Spain. Older adults after mild-moderate stroke without previous severe cognitive impairment or disability will be randomized into the control or intervention group (136 per group, total *N* = 272). The intervention group will receive 4 sessions of MI by trained nurses, including the design of a personalized rehabilitation plan agreed between stroke survivors and nurses based on stroke survivors´ goals, needs, preferences and capabilities. Main outcome will be the Functional Independence Measure (FIM). In-hospital physical activity will be measured through accelerometers and secondary outcomes using validated scales. The study includes a process evaluation and cost-utility analysis.

**Discussion:**

Final results are expected by end of 2020. This study will provide relevant information on the implementation of MI as a rehabilitation reinforcement tool in older stroke survivors. A potential reduction in post-stroke disability and dependence would increase person’s health-related quality of life and well-being and reduce health and social care costs. IMAGINE has the potential to inform practice and policymakers on how to move forward towards shared decision-making and shared responsibilities in the vulnerable population of older stroke survivors.

**Trial registration:**

ClinicalTrials.gov: NCT03434938, registered on January 2018.

## Background

Despite the positive advances obtained through acute treatments, stroke continues to be the leading cause of long-term disability in adults [1], reaching up to 40% of residual disability, and representing a huge burden for health and social systems, as well as for families [2].

Rehabilitation is crucial to reduce the gap between the persons’ disability and the demands of their environment [3]. In this sense, there are different interventions that focus on the different aspects of rehabilitation: some focus more on physical activity others on cognitive issues and some on regaining activities of daily living. Post-stroke rehabilitation entails a cyclical process, which includes: 1) identification of the person’s needs; 2) set of realistic and reachable goals; 3) implementing interventions according to these goals; and 4) follow-up, with potential revision of the goals and interventions [3]. Early rehabilitation [4, 5] and shared-goal setting between stroke survivors and healthcare professionals, based on the person’s preferences, values, previous routines and environment [6, 7], increase the odds of achieving better results [3]. Beneficial aspects include higher adherence to rehabilitation, increased personal satisfaction, shorter inpatient stays and greater goal attainment [6, 8]. This is also in line with the International Classification of Functioning, Disability and Health (ICF) [9], which promotes patient-professionals communication, and the terminology of which can be used to structure assessments, goals and plan interventions [10, 11]. However, patient participation in the process of goal setting is not standard clinical practice and even less so in geriatric rehabilitation, despite the fact that older persons need, due to their several comorbidities and various degrees of frailty, more guidance in defining their rehabilitation goals [12].

Further research has shown that psychological problems such as depression, apathy, anxiety, emotional and post-traumatic stress disorder are frequent complications following a stroke [13], reducing the motivation to engage in rehabilitation [13, 14]. Several studies have tested non-pharmacological interventions to reduce post-stroke psychological problems, such as cognitive-behavioral techniques and the improvement of social support networks [15, 16]. Nonetheless, most interventions are designed to be performed one month after the stroke and not during hospitalization in the acute phase, thus valuable time to begin and engage in rehabilitation is delayed [15]. Accordingly, new approaches are warranted.

Due to the population pyramid change and to the optimization of primary and secondary prevention treatments, the mean age of people with incident and fatal stroke has increased and is around 70.4 years old [2], [17]. Older adults have more frequently physical and mental comorbidities, and a pre-stroke reduced functional capacity that can be associated with social difficulties (i.e., living alone or with an old partner, low social support) which increase the risk of disability, institutionalization and death [18, 19]. Despite the high incidence of stroke in the aging population, older people with comorbidity and reduced functional ability have often been excluded from clinical studies precisely because of their high vulnerability and, therefore, it is necessary to increase research improving health outcomes in this segment of the population.

Motivational interviewing (MI) is a person-centered method where professionals and patients collaborate, utilizing a goal-oriented style of communication. This method gives particular attention to the language of change [20]. MI can be easily adapted to different healthcare settings and is known to strengthen personal motivation, empowerment, self-efficacy and commitment to specific personal goals by exploring and identifying the individual’s values and preferences [20]. MI has also demonstrated efficient in promoting physical activity, fostering treatment adherence [20, 21], and improving rehabilitation outcomes [20–22]. MI has been applied in frail older adults with mobility limitations, showing that it is safe and has a potential cost-effectiveness as a rehabilitative person-centered approach [23]. However, evidence of its effectiveness in older stroke survivors is lacking.

The IMAGINE study aims to investigate the effects of enriching usual geriatric rehabilitation with an adapted MI approach on different outcomes related to functional and clinical impact, persons’ satisfaction during hospitalization and cost-utility. We expect that the IMAGINE study will add a relevant contribution for the implementation of this intervention in older stroke survivors needing rehabilitation. Accordingly, this study should inform practice and policy makers on how to move forward towards shared decision making and shared responsibilities in a vulnerable population such as older stroke survivors [24]. With these aims, several hypothesis have been put forward:

1. MI, conducted during in-patient geriatric rehabilitation with older stroke survivors will result in an improvement in functional status at 30 days and 3 months, compared to standard geriatric rehabilitation alone.
2. The MI intervention will induce a statistically and clinically significant increase in physical activity during the stay in the geriatric rehabilitation department, and an improvement in physical performance, compared to controls.
3. The increase in physical activity and performance will be dose-correlated with the improvements in functional status.
4. The intervention will result in a more favorable and satisfying experience with care during hospitalization for the participants, caregivers and professionals involved.
5. Participants will increase their self-efficacy.
6. The intervention will be a cost-effective strategy to improve participant outcomes at an acceptable cost (or even with no relevant incremental costs).
7. The intervention will be safe for the stroke survivors. An increased number of adverse events potentially associated to geriatric rehabilitation and exercise will not be observed in the intervention group compared to control group

## Methods

### Design

Multicenter randomized clinical trial, with blinded outcome assessment.

### Setting

Geriatric rehabilitation units of three post-acute care hospitals in Catalonia, Spain.

### Population

Older stroke survivors admitted to one of the participating geriatric rehabilitation units. Inclusion and exclusion criteria are presented in Table 1.

**Table 1:** Inclusion and exclusion criteria

Inclusion criteria	Exclusion criteria
1. Older adult (> 60 years old) 2. Admitted to the geriatric rehabilitation unit after a mild-moderate stroke (ischemic or hemorrhagic, stroke severity at admission assessed by National Institute of Health Stroke Severity (NIHSS) scale < 17 points) 3. Able to provide informed consent, personally or by caregiver.	1. Previous diagnosis of dementia (ascertained from medical records) 2. Severe post-stroke cognitive impairment (Pfeiffer SMPQ> 7 errors) 3. Persistent delirium (> 7 days) after admission in the rehabilitation unit 4. Previous severe disability in activities of daily living (pre-stroke Barthel index < 20/100 points) 5. Severe stroke which might limit recovery (NIHSS> 16) 6. Aphasia or other problems hampering communication 7. Advanced and/or terminal condition (life expectancy not exceeding 6 months).

### Randomization and blinding

After inclusion in the study and obtaining informed consent, participants will be randomized centrally to intervention or control group in a concealed allocation manner. That means that, in each study site, a researcher not involved in the assessments will access the OxMaR minimization software through a secure web access [25], and obtain the participant’s random treatment allocation. Randomization will be based on the minimization method, which has proven to provide balanced allocation of study participants in small trials, with respect to prognostic participant characteristics [26]. For each participant, treatment allocation will partially depend on the characteristics of the already enrolled participants [25]. The prognostic factors included in the minimization algorithm are: study site, gender (male or female), and type of stroke (ischemic or hemorrhagic). Due to the nature of the intervention, participants cannot be blinded to allocation, but will be strongly advised not to disclose their allocation status at any assessments. The assessments will be conducted by a healthcare professional (physiotherapist) blinded to treatment allocation. The assessors are encouraged to maintain the blind as far as possible, nonetheless, the assessor must report if the participant allocation was revealed.

### Intervention

**Fig. 1:**
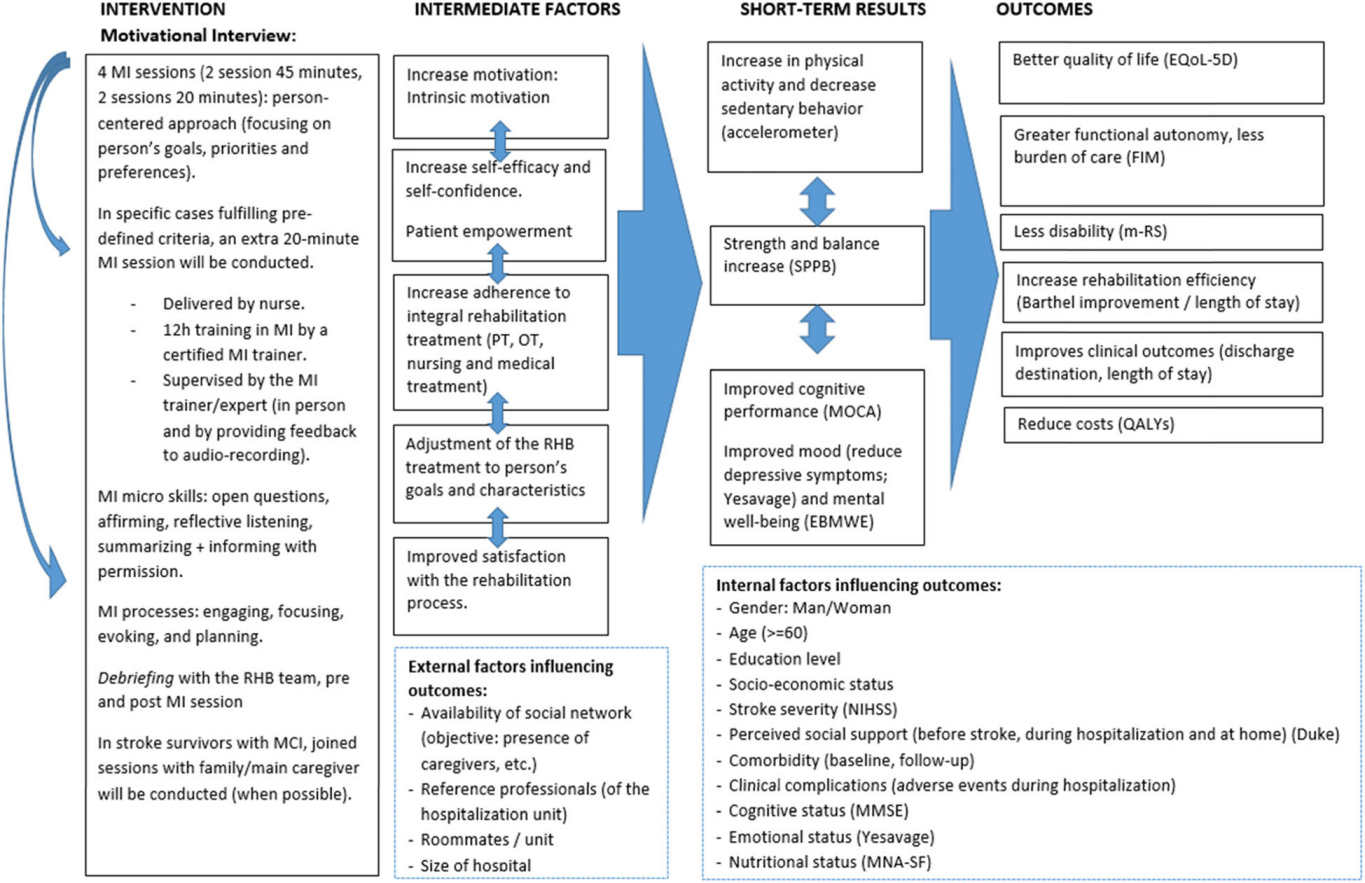
Logic model of IMAGINE project

The aim of the MI intervention, which will be integrated to the standard geriatric rehabilitation provided in the participating centers, is to empower, motivate and engage the person in planning and participating in the rehabilitation plan. The adaptation of the MI intervention is based on a previous pilot study carried out with older stroke survivors during geriatric rehabilitation, in one of the centers involved in the IMAGINE study [27]. Figure 1 shows the logic model of the intervention.

MI will be provided at the hospital ward, either in the person’s room or in an office nearby, according to their preferences and mobility. MI will be structured in four sessions: session 1 will be held one week from admission; session 2 within 6 days after the first session; session 3 at 1 week from the second session; and session 4 will be carried out pre-discharge.

The common goals for all sessions are:

1. To engage the stoke survivor in his/her care.
2. Collaborative co-creation of a personalized rehabilitation plan, which would complement the routine geriatric rehabilitation.
3. Reinforce engagement and adherence to the plan to maintain behavior change and functional improvement at 3 months.

**Fig. 2:**
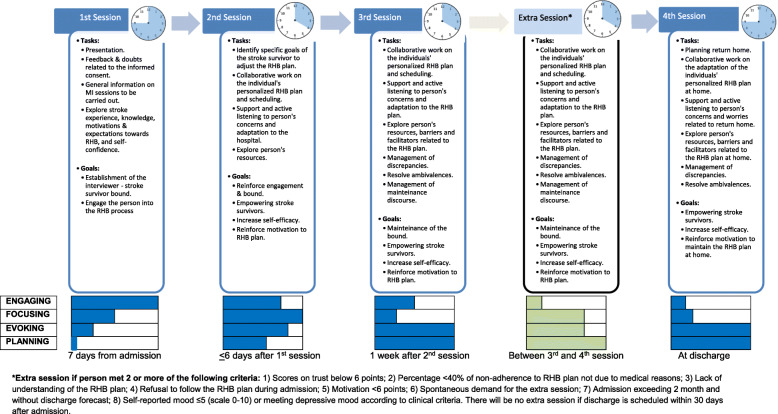
Structure and main contents of MI sessions

The emphasis on each of the different goals will vary according to each MI session (see Fig. 2). Although this intervention focuses on physical training and functioning, during the MI sessions people will be also encouraged to perform other recreational, socializing, relaxing or similar activities during hospitalization, always according to individual preferences and objectives.

MI involves the use of different core communication skills: 1) to ask open questions; 2) to affirm the person’s particular strengths, abilities, good intentions and efforts; 3) to use reflective listening; 4) to summarize the situation, and 5) to inform and advise with permission, which is useful to help people to reach their own conclusions about the relevance of any information the practitioner provides [20]. Each session will follow a semi-structured format to ensure homogeneity, while allowing tailoring. Content will generally include: 1) Creating engagement with the stroke survivor by exploring his/her preferences, values and goals, as well as his/her knowledge and expectations about stroke rehabilitation and recovery, 2) enhancing motivation by evoking strengths and abilities of the person, 3) follow-up and reinforcement, and 4) adapting the plan to the improved abilities and to home setting. As a practical milestone, during the second session the stroke survivor will establish tailored goals to complement standard rehabilitation, aimed at increasing involvement in self-care and daily living activities. When appropriate, in some cases debriefing with other professionals involved in the rehabilitation (physical therapists, occupational therapist or nursing staff) will be scheduled, to tailor and adjust the rehabilitation plan considering the functional situation. Figure 2 displays the structure of each session and specific criteria to conduct the additional interview between sessions three and four in case of external or internal modulators (which might have undermined the interviews). MI will be delivered by nurses trained through a certified MI course, and additional coaching by a psychiatrist who is a certified MI trainer will be offered to them throughout the intervention period (two group meetings per year and on demand individual sessions). During the study, quality control of the MI sessions using Motivational Interviewing Treatment Integrity (MITI) Code 3.1.1 [28] will be performed through video recording of a random selection of sessions.

### Control group

The control group will receive standard geriatric rehabilitation. Routine geriatric rehabilitation in post-acute centers in Catalonia is homogeneous in terms of process and outcome indicators and general staff/patients ratios are marked by the Department of Health [29]. Standard geriatric rehabilitation includes a multidisciplinary and individualized rehabilitation plan, which incorporates physiotherapy, occupational therapy and speech therapy. This plan is established after a comprehensive multidisciplinary geriatric assessment, and shared through the electronic health records.

### Outcome assessments

All outcome assessments will be performed by trained physiotherapists, certified to use the Functional Independence Measure (FIM) and blinded to treatment allocation. Table 2 summarizes assessment tools administered in this study and the specific assessment time periods of each one. We will monitor and record whether group allocation is revealed during the outcome assessment.

**Table 2:**
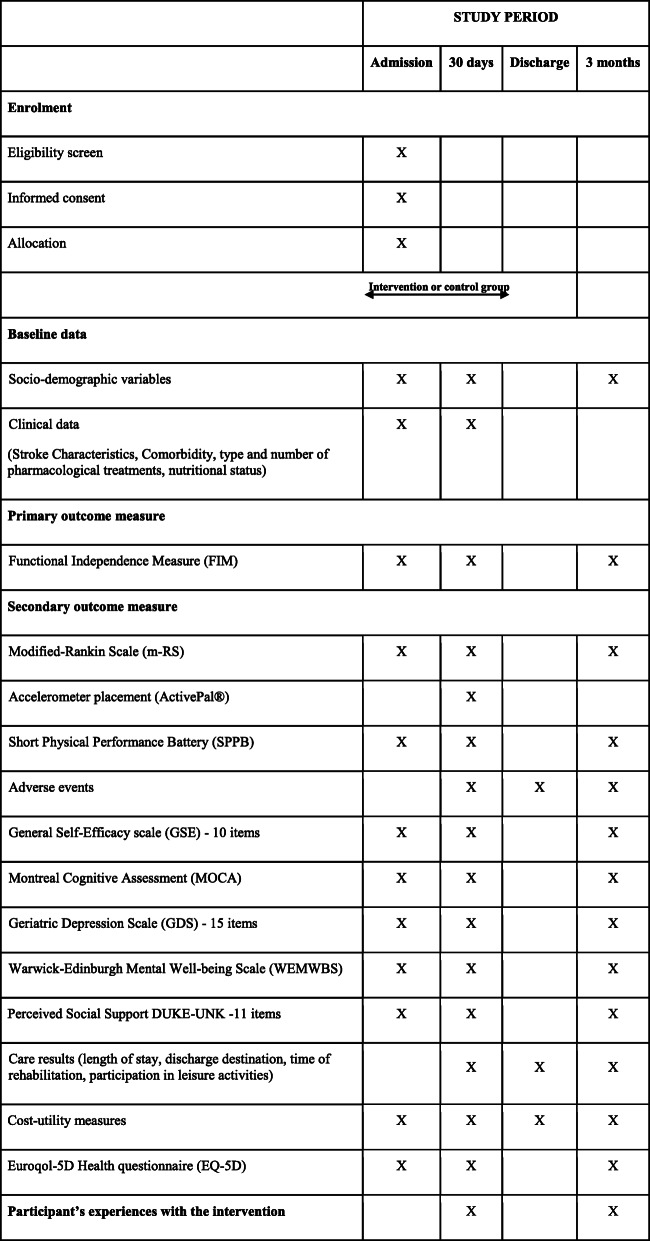
Schedule of enrolment, interventions, and assessments for the IMAGINE study

#### Primary outcome measures

Functional Independence Measure (FIM), validated for use in stroke survivors [30], is a sensitive and comprehensive tool to assess the level of independence in basic activities of daily living, which is considered the golden standard for stroke rehabilitation. The scale includes 18 items, grouped into 2 subscales: 1) motor and 2) cognition. Each item is scored on a 7-point ordinal scale (1–7, higher values indicating more independence). Combined motor (13–91 points) and cognitive (5–35 points) subscale scores provide the total score (18–126 points; higher values indicating more independence). In this study, a version translated into Spanish was administered.

#### Secondary outcome measures

- Complementary measures of function/disability. The Modified-Rankin Scale (m-RS) is a short measure of disability and dependence, routinely used for 3 months clinical follow-up, and rated in an ordinal scale with 6 categories ranging from zero (no symptoms) to five (complete physical dependence). A seventh category can be added to signify death [31]. In this study, a version translated into Spanish was administered.
- In-hospital physical activity will be measured once through accelerometers, placing ActivPAL® devices on the preserved leg during 7 consecutive in-hospital days, to measure mainly time spent sitting and standing. This device has been validated in post-acute stroke samples [32, 33].
- Physical performance improvement will be measured using the Spanish validated version of the Short Physical Performance Battery (SPPB) (0–12 points, worse–best), which includes sub-tests of balance, strength and gait speed [34].
- Adverse events registration will include: falls, fractures, cranial traumatism, pain (Visual Analogue Scale from 0 to 10 points), cardiovascular events (angina, myocardial infarction, TIA, stroke), aspiration pneumonia/respiratory infections, readmissions to acute hospitals and death.
- General Self-Efficacy scale [35] is a 10-item tool designed to assess optimistic self-beliefs to cope with a variety of difficult demands in life. This scale is directly correlated to emotion, optimism and work satisfaction. The total score ranges between 10 and 40, with a higher score indicating more self-efficacy. Negative correlations are found with depression, stress, health complaints, burnout, and anxiety.
- Montreal Cognitive Assessment (MOCA) [36] is a brief instrument recommended for cognitive impairment (CI) screening in people who have suffered a stroke or TIA. It is sensitive to changes in acute temporary CI after mild stroke/TIA [37]. The test assesses visuospatial abilities, short-term memory, executive functions, language and fluency, abstraction thinking, orientation, attention, concentration, and working memory. The scale includes 10 questions (total score range 0–30). Higher scores indicate higher cognitive performance.
- Mental health: Depressive symptoms by means of the Yesavage Geriatric Depression Scale (GDS) 15 items (0–15 points; worse – best) [38] and the mental wellbeing with the Warwick-Edinburgh Mental Well-being Scale (WEMWBS), which includes 14 questions with answers coded from 1 ‘Never’ to 5 ‘Always’ (14–70 points, worst – best) [39].
- Duke Social Support Index 11 items (DUKE-UNK-11) will be used to assess social support and effective social contacts during the rehabilitation process, effective caregiver at home, and perceived social support (11–55 points; lower – higher social support) [40].
- Care results. We will collect data on length of stay (days), discharge destination (home, nursing home, long-term care, acute hospital, death), and hours of rehabilitation.
- Participation in leisure activities during admission and after-discharge, reported by nurses, stroke survivors and / or caregivers, will be collected.
- Rehabilitation efficiency. Scores will be computed in terms of improvement of FIM/length of stay.
- Quality of life. A generic measure of quality of life will be obtained using the EQ-5D-5L tool, which includes two components. A health state description comprises 5 items assessing the dimensions of mobility, self-care, usual activities, pain/discomfort, and anxiety/depression and evaluation, via categorical questions. An evaluation part using a Visual Analogues Scale for retrieving from respondents an overall judgment on their health in a scale from 0 to 100 [41, 42].

#### Covariates assessed at admission

- Socio-demographic variables: age, gender, civil and marital status.
- Clinical data: Stroke characteristics (ischemic/hemorrhagic, Oxfordshire CSPC, acute treatment, etiology) clinical variables such as chronic diseases and comorbidity measured by a Spanish translated version of the Charlson index [43] will be collected. In addition, type and number of pharmacological treatment, nutritional status with the Spanish validated version of the Mini Nutritional Assessment-Short form (MNA-sf) [44].

### Procedure

Table 2 depicts full procedures of the project.

In case of discharge before 30 days, the follow-up assessment will be performed the day of discharge, always registering the date of the assessment. In case of unplanned discharge (e.g. to emergency/acute hospital), we will collect at least routine available data collected in the rehabilitation unit and administrative data. The 3 months assessment will be performed either at an outpatient’s clinic or at the participant’s home. If face-to-face follow-up is not possible, a telephone assessment can be conducted. In all cases, assessment will include at least the telephone version of: Barthel, Rankin, Lawton, FIM, FAC, GSE, DUKE-UNK-11, GDS-15, EQ-5D.

### Process evaluation

Since MI intervention is a complex intervention, as observed in Fig. 1 (logic model), the trial includes a process evaluation to enhance the interpretation of the results of effectiveness. This is based on the Medical Research Council (MRC) guidance [45, 46], and focuses on how the context, implementation and impact mechanisms might influence each other and shape outcomes. Specifically, we will assess: 1) the quantity and quality of what is delivered (implementation); 2) the generalizability of the effectiveness by understanding the role of context and the comparability between intervention sites; 3) the mechanisms of impact; and 4) perceived impacts and adverse events.

Each assessment will be addressed complementarily by quantitative and qualitative methodological procedures. Observation checklists and attendance registry will be conducted after each MI session by the interviewer. These procedures will collect information on fidelity (achievement of goals and tasks of each session), dose and adverse events. Standardized scales (i.e. self-efficacy) will be included in the baseline, post-intervention and follow-up assessments to explore mechanisms of impact according to the logic model. Contextual characteristics will be collected in a semi-structured questionnaire to capture shared and specific features of each intervention site.

Complementarily, a qualitative evaluation will be used to explore participant experiences with the implementation of the intervention, their personal and social context, the mechanisms of impact and their perceived effects as well as unexpected events. It will consist in in-depth interviews at 30 days and at 3 months on a purposeful sample of participants, selected to maximize variability by gender, disability levels, socio-economic levels, and social support. In addition, a purposeful sample of caregivers and professionals who performed the intervention will be also interviewed. Finally, a focus group with reference rehabilitation professionals of the units will be conducted in each intervention site.

The process evaluation will be done by a MD and a social worker with great experience in qualitative analysis, designing complex evaluations and process evaluation. These professionals will not have another role in this project. The results will be reported at completion.

### Sample size calculation & statistical analysis

Sample size calculation is based on the results of previous RCTs assessing the effect of geriatric rehabilitation on improvement of the FIM. Accordingly, it is expected that geriatric rehabilitation will induce a 20 points improvement in FIM between admission and discharge [47]. This change has been established as “minimal clinically meaningful” improvement [48]. It is expected that MI plus geriatric rehabilitation will result in a “moderately relevant” clinical improvement in FIM, estimated to be 30 points. That is, MI will induce an additional improvement of 10 points (which corresponds to approximately 10% of total FIM score), compared to the control group. Considering a common standard deviation of 27.9 points in FIM, a 10% of drop-out rate at 30 days, and accepting an alfa risk of 0.05 and a beta risk of 0.2 in a bilateral contrast, we would need to include 136 stroke survivors in each branch (total sample = 272) to detect this improvement as statistically significant. Calculations were obtained using the GRANMO program [49].

Descriptive statistics will be presented for the overall characteristics of the study sample (intervention and control group) as frequencies and percentages for nominal or categorical variables and means and standard deviations for continuous ones. Baseline comparisons between treatment groups will be assessed as t-tests and chi-square tests, to check for potential imbalances in group composition and characteristics.

The effect of MI in the change in total FIM score between baseline and follow up (30 days) will be explored through a longitudinal repeated-measures mixed effects linear model, with the total FIM score as dependent variable, and a 2-level fixed factor accounting for the treatment group (MI or control). The model will adjust for clinically relevant covariates, such as age, gender and the amount of physical activity.

Group analyses will be carried out on an intention-to-treat principle. Missing data will be replaced by multiple imputations, after conducting explorations of the possible missingness mechanisms.

Cost-utility analysis will be also performed to compare the benefit and costs of a MI programs. Cost-utility will be measured as the ratio between direct costs (workforce time use to deliver the MI intervention and the related training and supervision, supplementary tests, use of other hospital resources, medical visits, readmissions, treatments, ambulatory rehabilitation, etc.) during hospitalization and also post-discharge (at 3 months) and Quality Adjusted Life Years (QALYs). QALYs will be derived using the EQoL-5D before and after the treatment and at 3 months. Therefore, cost-utility will be calculated as the incremental ratio in € for 1 QALYs in the intervention vs. control group [50, 51].

All analyses will be carried out in Stata 15 (StataCorp. 2017. *Stata Statistical Software: Release 15*. College Station, TX: StataCorp LLC).

### Legal and ethical aspects

The protocol and the template informed consent forms has been approved by the Ethics Committee on Animal and Human Research (CEEAH) of the Universitat Autònoma de Barcelona (reference number CEEAH 3715). The rest of the participating centers’ Ethics Committees ratified these premises. At the beginning of the project, an advisory board will be created including stroke survivors’ and caregivers’ representatives (from dedicated Associations or from the participating institutions), health and social care professionals and policymakers, which will meet periodically with the research team. The commission of this board will be to: 1) support and give feedback about the refinement of the study and intervention design; 2) promote constant feedback on project progress and help to overcome potential barriers; 3) foster communication, dissemination and knowledge translation.

Prior to any assessment and intervention, all participants in the study granted written and verbal informed consent.

All study-related information will be stored securely at the reference center Server. All participant information will be stored in locked file cabinets in areas with limited access. All reports, data collection, process, and administrative forms will be identified by a coded ID number only to maintain participant confidentiality. All records that contain names or other personal identifiers, such as informed consent forms, will be stored separately from study records identified by code number. All local databases will be secured with password-protected access systems. Forms, lists, logbooks, appointment books, and any other listings that link participant ID numbers to other identifying information will be stored in a separate, locked file in an area with limited access.

The coordinator of each intervention site will be given access to the cleaned data sets, housed on the file transfer protocol site created for the study, and all data sets will be password protected. Project Principal Investigators will have direct access to their own site’s data sets, and will have access to other sites data by request. To ensure confidentiality, data shared with project team members will be completely anonymized without any identifying participant information.

The feasibility and scientific quality of the trial has been peer-reviewed and approved by the Principal Investigators and Research and Development team. The trial protocol will permit its reporting in line with the Consolidated Standards of Reporting Trials (CONSORT) guidelines [26]. The Standard Protocol Items: Recommendations for Interventional Trials (SPIRIT) checklist [52, 53], and the Template for intervention description and replication (TIDieR) checklist [54] are both provided as additional files 1 and 2.

## Results

**Fig. 3:**
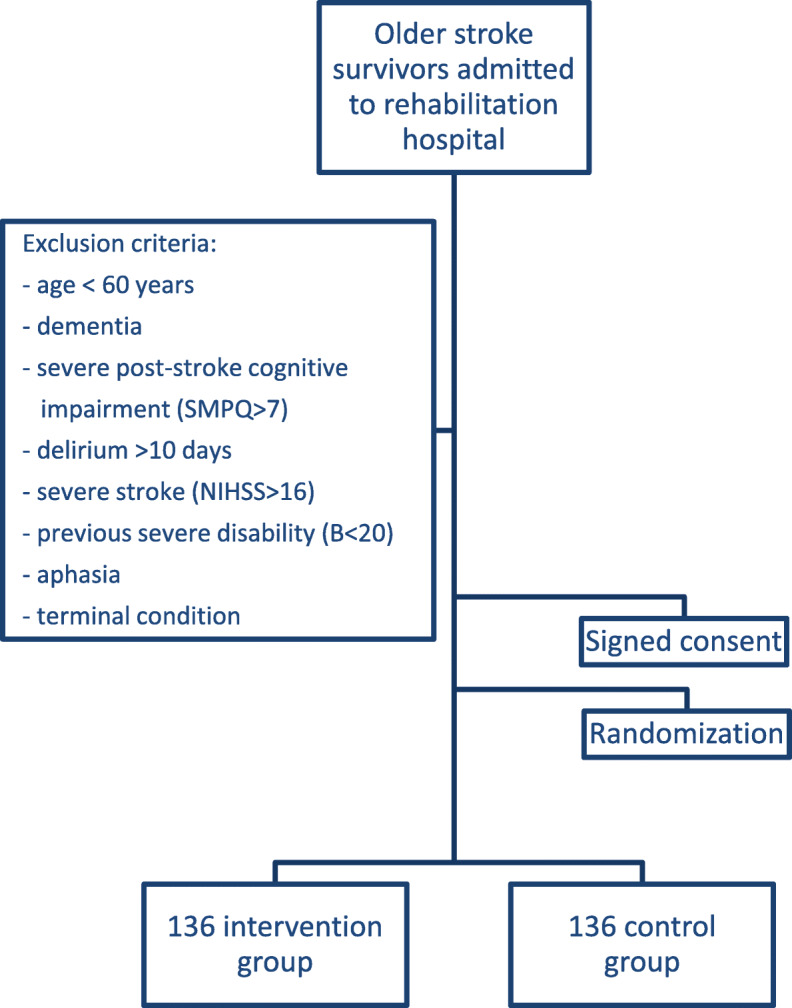
Flow chart

Final results are expected by end of 2020. See flow chart (Fig. 3).

## Discussion

This project is expected to provide new evidence on the impact of MI on older stroke survivors, increasing the understanding on the implementation of a non-pharmacological intervention based on a person-centered approach, and assessing its impacts on objective outcomes. The project will be performed from a “triple aim” viewpoint (healthcare, efficiency and person-centered outcomes and experiences), through multi quantitative and qualitative methods. Accordingly, the IMAGINE study will develop a new area of research with an innovative approach: empowering older stroke survivors that are in vulnerable conditions, and building their rehabilitation process with them rather than only for them. Thus, it changes the person’s perspective from his/her limitations to his/her strengths. In the same line, this study focuses on a novel approach by fostering residual capacities based on personal goals instead of “treating disability”, in line with novel frameworks [10, 55]. The MI approach can easily be part of professional training or configure novel specialty training for healthcare professionals involved in rehabilitation services, providing a unique opportunity of scaling up the intervention. The focus on person centered care is expected to increase person satisfaction, which will be assessed by exploring the experiences of the stroke survivors and professionals involved. In addition, the trial is based on objective outcomes that are relevant for older stroke survivors, since mobility and physical function limitations are a major barrier for quality of life. We will also assess other aspects such as well-being or self-efficacy, as a health resource with major influence on quality of life and with potential to explain the mechanisms of impact of the MI intervention.

Regarding stroke survivors, MI has been used mainly in chronic stages in order to promote and maintain pharmacological treatment and lifestyle changes [32, 56, 57]. Recently, two research groups have described a possible association between the early use of MI, functional and mood improvement and a reduction of mortality [58, 59]. However, as none of these studies focused on older adults, the evidence of its use during the subacute phase of stroke in older adults is scarce [60]. Similarly, English et al. [32] assessed the feasibility of MI in a small sample (*N* = 35) of stroke survivors (> 6 months) in the community. They also assessed the impact on physical activity, as the reduction of sitting time measured using accelerometers. The intervention was found feasible and safe but the observed sitting time reduction was not significantly different from the control group. Recent studies on frail older adults with mobility limitations from the community have confirmed safety and showed potential cost-effectiveness of a rehabilitative person-centered approach [23]. These results are in line with other studies that suggest cost-effectiveness [23, 61], and cost-utility [62, 63] of MI used to promote changes in lifestyle, in different settings and populations. However, such studies have not been carried out with stroke survivors.

Potential critical aspects and risks might be an insufficient sample size or important attrition; although in case of need, other geriatric rehabilitation centers of the Catalan healthcare network (Catsalut) could be included. Strengths of the study are the three intervention sites, referent centers for geriatric stroke rehabilitation in Catalonia, who have also previously shown their recruitment capability. Moreover, our previous MI pilot [27] showed high acceptance of the intervention, which should reduce the risk of dropouts. Although in a non-pharmacologic trial it is almost impossible to blind participants and professionals delivering the intervention, procedures have been set to guarantee as much as possible blinding outcome assessors.

This project has the potential to directly change the decision making process in the rehabilitation of older stroke survivors. As almost half of stroke survivor’s experience relevant residual disability, with stroke being the main cause of disability in Western countries [2], new rehabilitation and recovery strategies are needed and will have a high impact for the society. Likewise, the knowledge gained by this project, including the intervention and its effects also in terms of costs, should inform current health care policies at national and international level on how to enhance established rehabilitation programs with an empowering approach, thus taking a major profit of currently limited economic and human resources.

## Supplementary information

**Additional file 1.**

**Additional file 2.**

## Data Availability

Data sharing is not applicable to this article as no datasets were generated or analysed for this study protocol.

## Abbreviations

FIM: Functional Independence Measure
ICF: International Classification of Functioning, Disability and Health
MI: Motivational interviewing
MITI: Motivational Interviewing Treatment Integrity Code
m-RS: Modified-Rankin Scale
NIHSS: National Institute of Health Stroke Severity
QALY: Quality Adjusted Life Years
